# Supplementary material for: Transcriptomics of Improved Fruit Retention by Hexanal in ‘Honeycrisp’ Reveals Hormonal Crosstalk and Reduced Cell Wall Degradation in the Fruit Abscission Zone
Source: Int J Mol Sci. 2021 Aug 17;22(16):8830. doi: 10.3390/ijms22168830 (PMC8396267; doi:10.3390/ijms22168830)
Supplement: Supplementary file 1 [file ijms-22-08830-s001.zip › Supplementary material Tables S1 S7 and Figures S1 S2.pdf]

Table S1: Summary results of the RNA-Seq run

**Data**	**Control_rep1**	**Control_rep2**	**Hexanal_rep1**	**Hexanal_rep2**
RNA concentration (ng/μL)	428	466	381	409
Read length (bp)	150	150	150	150
Number of raw reads	25,615,611	20,059,664	19,717,528	33,233,141
Failed reads during quality and clipping	125,947	95,215	85,558	154,401
Reads passed initial quality	25,489,664	19,964,449	19,631,970	33,078,740
Shorts reads	111,506	83,888	74,314	135,340
rRNA	406,755	194,188	450,722	1,058,587
Total mappable reads	25,239,628	19,846,108	19,360,138	32,444,090
Reads mapped to genome	24,133,526	19,084,392	18,796,051	30,977,816
Not mapped to genome	1,338,387	967,217	754,635	1,779,053
% of reads mapped to genome	95.62	96.16	97.08	95.48
Gene sense count	20,763,558	16,585,294	16,152,345	26,269,328
Number of detected genes at >1 raw read	35,046	34,394	34,845	35,042

rep1 represents the RNA extracted from the fruit-AZ harvested from commercial orchard located in Site A, whereas rep2 from commercial orchard located in Site B.

Table S7: Forward and reverse primer details of the selected genes in the ethylene biosynthesis and signalling pathway and cell-wall re-modelling.

No	Gene	Gene ID (*Malus domestica*)	Forward primer	Reverse Primer
1	SAM2	MD13G1141700	TCAACCCAGCACGATGAGAC	TGAGCACCCCATCCTCCATA
2	ACO3	MD09G1114800	GCTGCTGGACTTGTTGTGTG	TGGAAGAGCAAGATGAGGCC
3	ETR2	MD13G1209700	AGCATGGCACTTGTTCTTCG	CTCGAGCATTTTCCGCATCA
4	ERF17	MD15G1221100	GAAGCAGACGATTGGGAAGC	TCGGGGAAATTAAACTTGGCA
5	EXPA6	MD03G1090700	GTGAGAGTGTTTGAGGGGCA	TGGTGCGGGCTACAAATTCT
6	EXPA8	MD07G1233100	GCTGCGGGTCTTGTTATGAG	CTGAAGGAGACGGGGACAAT
7	EG19-*like*	MD06G1105900	TAGCTGATAAACCACCGCAC	CAAGTGACTCTCTGGTTGGG
8	1,4-β-EG3	MD10G1003400	GAGGCCCGAAGATATGGACA	ATCACTGTATGCACCTCGGT
9	MdAct	XM_008362405.3	GTGGATTGCAAAGGCAGAGT	CATAATTTGCTCGCCTCCAT
10	MdHis3	AY347801.1	TGGAACTGTTGCTCTTCGTG	CTCAAACAACCCGACAAGGT

Sequencing details to the gene set 1-8 were obtained from Genomic Database for Rosaceae and 9-10 from NCBI data base.

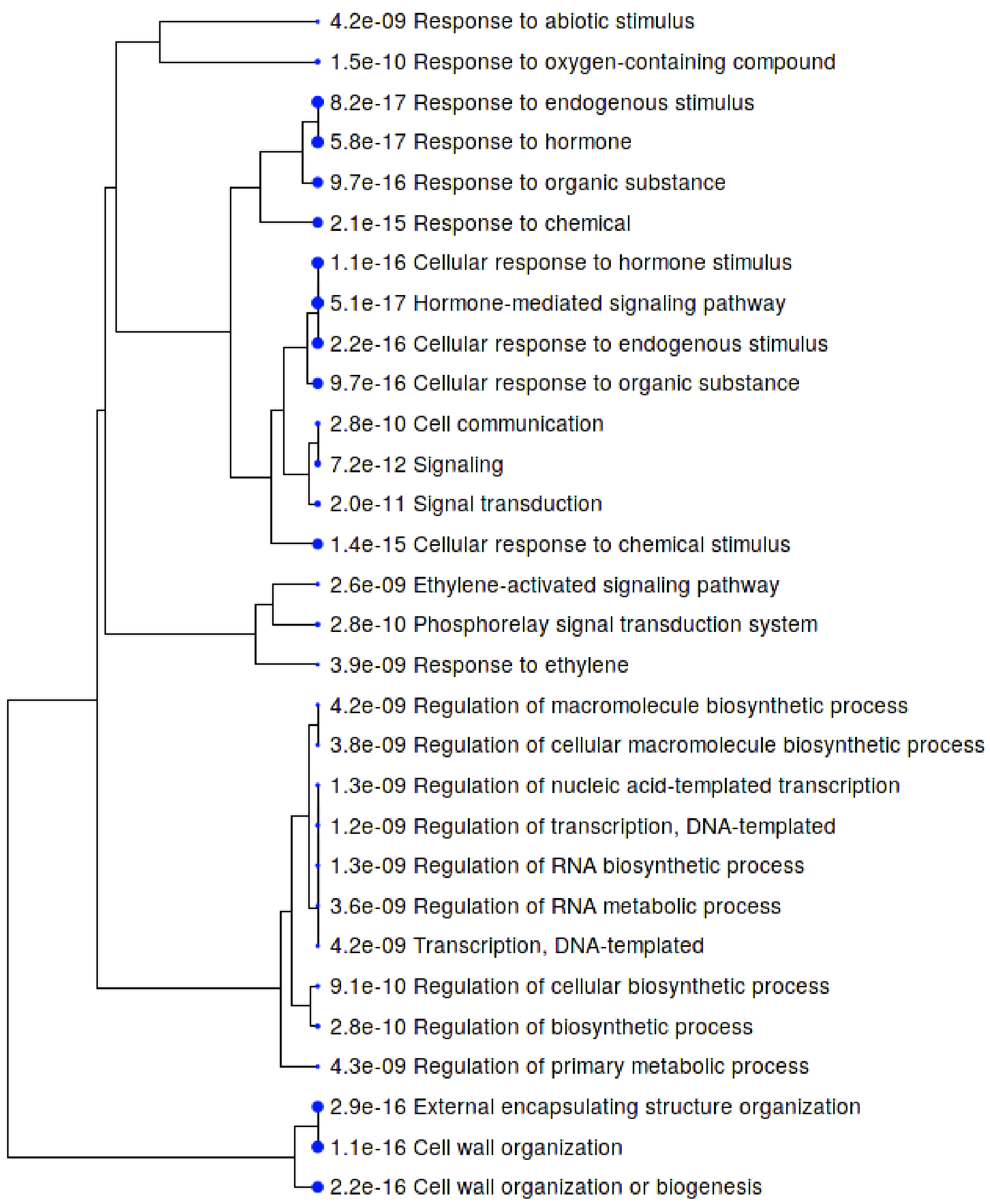

Fig. S1: Hierarchical clustering tree summarizing the first 30 enriched functional categories belonged to BP (FDR, $P < 0.01$). Pathways with many shared genes are clustered together. Bigger dots indicate more significant P-values. Additional information on the functional pathways is presented in the supplementary information Table S4

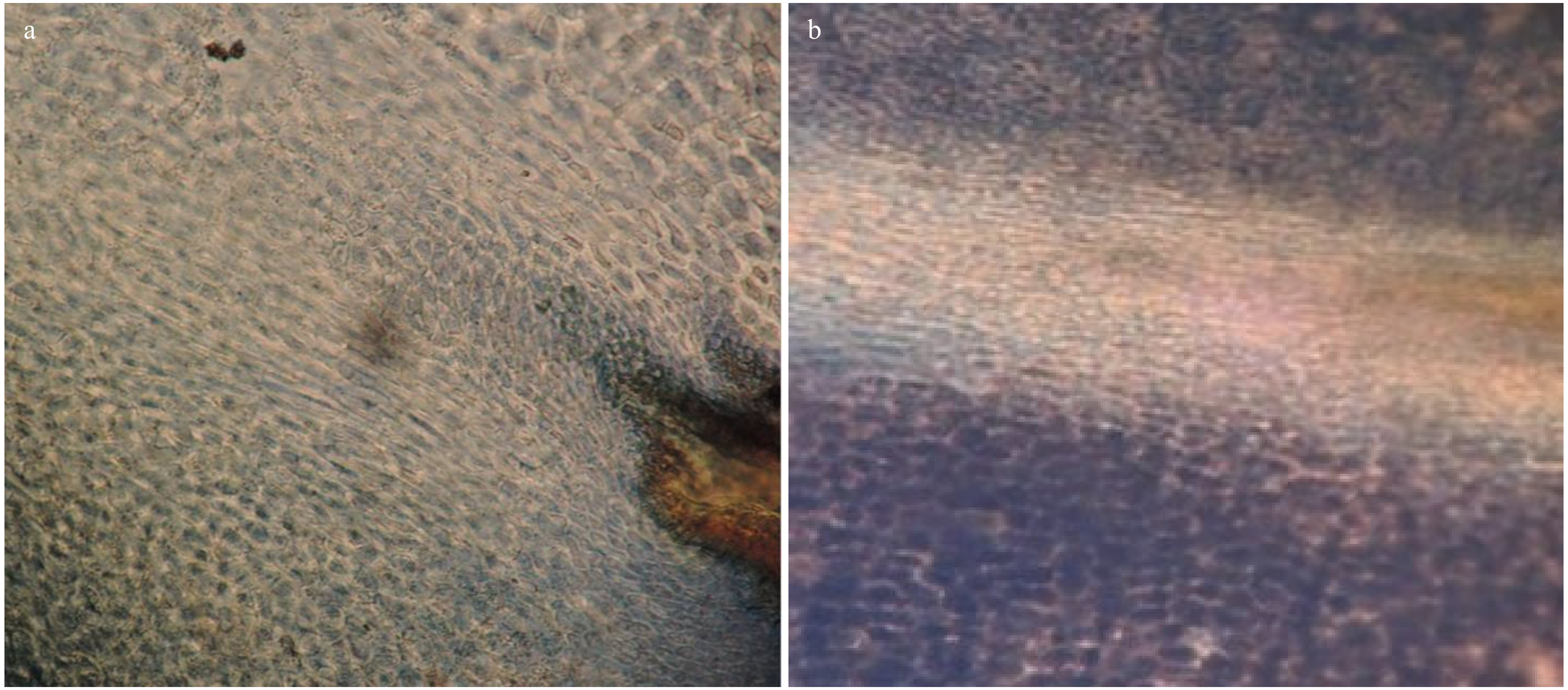

Fig. S2: Compound microscopic view of fruit-AZ cells sampled at the end of the fruit retention study (49 days after harvest). (a) AZ-cells from control fruits and (b) shows the AZ-cells from the hexanal treated fruits. Lactophenol cotton blue dye was used to stain the AZ tissues. Treated fruits AZ layers more organized with more defined horizontal layers that stained better than control
